# Is lineage decision-making restricted during tumoral reprograming of haematopoietic stem cells?

**DOI:** 10.18632/oncotarget.6145

**Published:** 2015-10-19

**Authors:** Geoffrey Brown, Isidro Sanchez-Garcia

**Affiliations:** ^1^ School of Immunity and Infection, College of Medical and Dental Sciences, University of Birmingham, Birmingham, UK; ^2^ Experimental Therapeutics and Translational Oncology Program, Instituto de Biología Molecular y Celular del Cáncer, CSIC/Universidad de Salamanca, Campus M. de Unamuno s/n, Salamanca, Spain; ^3^ Institute of Biomedical Research of Salamanca (IBSAL), Salamanca, Spain

**Keywords:** haematopoiesis, leukemia

## Abstract

Within the past years there have been substantial changes to our understanding of haematopoiesis and cells that initiate and sustain leukemia. Recent studies have revealed that developing haematopoietic stem and progenitor cells are much more heterogeneous and versatile than has been previously thought. This versatility includes cells using more than one route to a fate and cells having progressed some way towards a cell type retaining other lineage options as clandestine. These notions impact substantially on our understanding of the origin and nature of leukemia. An important question is whether leukemia stem cells are as versatile as their cell of origin as an abundance of cells belonging to a lineage is often a feature of overt leukemia. In this regard, we examine the coming of age of the “leukemia stem cell” theory and the notion that leukemia, like normal haematopoiesis, is a hierarchically organized tissue. We examine evidence to support the notion that whilst cells that initiate leukemia have multi-lineage potential, leukemia stem cells are reprogrammed by further oncogenic insults to restrict their lineage decision-making. Accordingly, evolution of a sub-clone of lineage-restricted malignant cells is a key feature of overt leukemia.

## INTRODUCTION

An important question regarding stem and progenitor cells choosing a developmental route is whether the routes these cells follow to a mature cell type are invariant or versatile. By versatility, we mean the extent to which there are alternative pathways to a cell type and whether cells that have developed part of the way along a lineage pathway can be diverted along a different lineage pathway. In this case, the alternative pathway fate options might be viewed as clandestine. Overall the question is one of mutability of cell fate: in other words, the extent to which cells can readily reprogram their lineage potential(s). In 1987, Shankland described flexibilities to developmental pathways in the embryo of the leech [1]. Cells can use alternative pathways to give rise to an end cell phenotype and this versatility can also be latent. Neurons and epidermal specifications normally descend from the ‘p blast’ group of precursors, but when the ‘p blast’ lineage is ablated the ‘o blast’ cells then give rise to neurons and epidermal cells. This adaptation of the ‘o blast’ cells to a new pathway means that lineage pathways are open to reprogramming during normal leech development.

Strikingly similar findings have emerged from recent studies of mouse stem cells. Studies by Chan and co-workers have shown that quite unexpected cell types can alter their lineage fate [2]. Cells residing within the femoral growth plates of bone and that express the haematopoietic cell surface marker Tie2 are not able to form bone, cartilage or stroma. Adipose tissue contains high numbers of Tie2^+^ cells and collagen sponges containing recombinant bone morphogenic protein 2 (BMP-2) were inserted into the inguinal fat pad of a reporter mouse (Tie2Cre x MTMG) that labels Tie2 cells with GFP and other cells with RFP. Ossicles provoked by BMP-2 and harvested a month later contained Tie2-derived (GFP positive) and Tie2-negative-derived (RFP positive) osteocytes, indicating both these cell populations had undergone a shift to a skeletal fate. The use of a parabiont model (actin-GFP transgenic/non-GFP congenic) showed that circulating cells had not contributed to the BMP2-driven formation of ectopic bone. In essence, BMP-2 appears to have reprogrammed Tie2^+^ and Tie2^−^ cells resident in the extra-skeletal site, inferring flexibility in the programme status of these cells. These data also highlight the importance of environmental niches to the regulation of choice of lineage pathways.

### The heterogeneity and versatility of haematopoietic stem and progenitor cells

The mammalian blood cell system provides developmental biologists with one of the best models for unravelling how a stem cell – the haematopoietic stem cell (HSC) – gives rise to the many different types of cells of the blood and immune systems. Information gained about the nature of cell compartments to developmental progression led to definition of the cellular origins of the various types of leukaemia. The haematopoietic and skeletal systems have a similar architecture, adhering to a multi-potent stem cell giving rise to a variety of well-compartmentalised down-stream progenitors that are becoming progressively restricted in their lineage capacities. In recent years, our viewpoint on the architecture of haematopoiesis has changed to take into account the idea that haematopoietic stem and progenitor cells (HSPC) are more versatile than previously thought [1, 3, 4].

**Figure 1:**
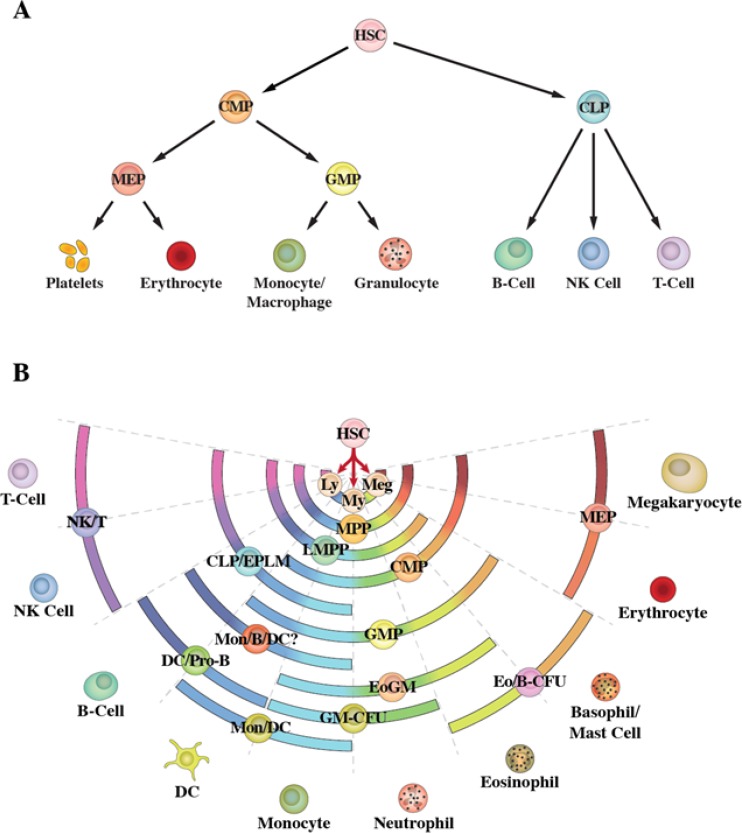
Schematic representations of haematopoiesis **(A)** Depicts the classic model in which the haematopoietic stem cell makes an irrevocable choice between the myeloid and lymphoid pathways. **(B)** Depicts the pair-wise model. Differentiation options are envisaged as a series of invariant pair-wise developmental relationships with cells becoming gradually biased towards producing one cell type or another.

Since the 1980s, the model generally used to describe haematopoiesis is an invariant cell lineage tree for developing progenitor cells. The tree depicts a series of binary decisions [5]. The first of these is the HSC makes an immediate, and irrevocable, choice between the myeloid and lymphoid pathways of development, as evidenced by the existence of a common myeloid progenitor (CMP) and common lymphoid progenitor (CLP) (Figure 1A) [6, 7]. CMP and CLP follow preferred routes to a particular cell fate; with these evidenced by colony-forming cells (CFC) which give rise to certain types of mature cells when bone marrow cells are dispersed in semi-solid medium. These include, colonies containing a variety of cell types, for example, granulocyte/erythrocyte/megakaryocyte/macrophage-CFC. Colonies containing only two types of cells such as granulocyte/monocyte-CFC identify a bipotent granulocyte/monocyte progenitor cell (GMP).

Whether the ‘classic’ model is an accurate depiction of haematopoiesis has been questioned by the identification of sub-groups of haematopoietic progenitor cells (HPC) with lymphoid potentials and only a sub-set of myeloid potentials. These cells include early progenitors with lymphoid and myeloid potential (EPLM) [8] and lymphoid-primed multipotent progenitors (LMPP) [9] which have lymphoid potentials and myeloid potentials excluding those for erythroid and megakaryocyte development. The identification of EPLM and LMPP excludes a strict schism as to separate programming of myeloid and lymphoid fates as HSC do not pass through an obligatory CMP *versus* CLP phenotype [10] Whilst EPLM and LMPP preclude this strict dichotomy they do not contravene the clear existence of CMP and CLP. Instead, the main point is that the combination of partial myeloid fates and lymphoid fates within EPLM and LMPP span the fate potentials of CMP and CLP, respectively. To extend this notion, there might well be a plethora of intermediate HSC-derived HPC with different combinations of differentiation options. The options available to HPC have been revealed using *in vitro* clonogenic assays and by the extent to which cell lines that typify immature cells can be manipulated by culture conditions (growth factors, retinoids and phorbol ester) to differentiate along various pathways [reviewed in 3]. Indeed, there is a striking array of progenitors and cell lines with different combinations of lineage options. To add to this variability, many of the known HPC that are viewed as homogenous might be an admixture of cells and markers are lacking to resolve sub-populations. The use of a combination of markers and of *in vivo* assays has revealed HSC to be a heterogeneous population of cells: HSCs that are platelet-, myeloid- and lymphoid-biased have been described [11–14].

In addition to sub-dividing HSC, the presence of lineage biases within these cells brings to attention two interesting notions. First, the biases having originated in HSC might be presumed to persist in their progeny. In keeping with this, new markers and combinations of markers are likely to reveal such heterogeneity within HPC. Second, HSC appear to display a developmental propensity to differentiate readily and irrepressibly diversify and differentiate. In fact, they do so when cultured with appropriate growth and survival factors and, as considered later, some of these growth factors instruct fate adoption.

Whilst fate options occur in varied combinations there is order to the sets of fates available to individual cells. Our own viewpoint on haematopoiesis is to not draw strict lines representing routes from HSC *via* their progeny to end cell types. The pair-wise model shows a series of invariant pair-wise developmental relationships, with the fate choices available to HSC as a continuum [10] (Figure 1B). The ordering of near-neighbours relates to the sets of potentials available to various known oligopotent HPC [reviewed in 3, 10], as represented by the arcs in the figure. The pair-wise model accommodates lineage-biased HSCs that are platelet-, myeloid- and lymphoid-biased which are also interesting as to targets for transformation in leukaemia.

### Multiple routes, clandestine options and lineage reprogramming

**Figure 2:**
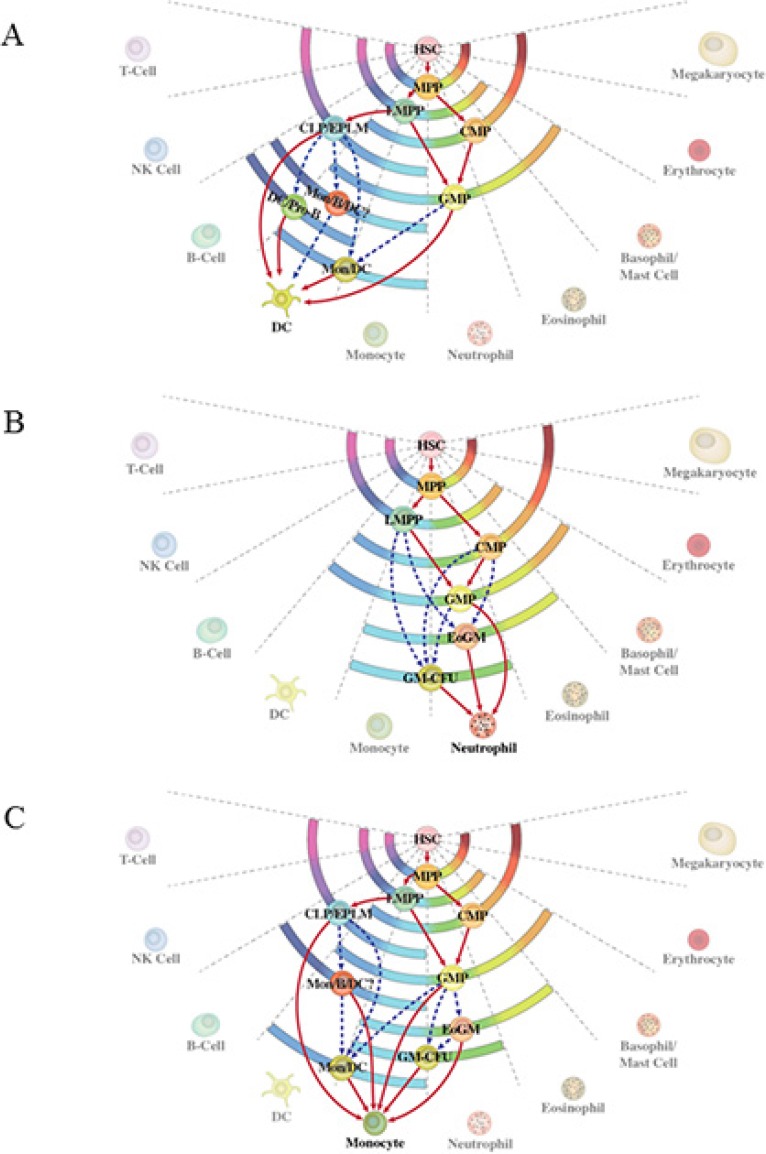
Alternative routes to dendritic cells, neutrophils and monocytes **(A)** The alternative routes to dendritic cells relate to the generation of these cells from either the common myeloid progenitor or the common lymphoid progenitor. **(B)** and **(C)** Routes to neutrophils and monocytes are either known routes from studies of progenitor cells (solid lines) or inferred by virtue of which known progenitors are or are not able to give rise to one another regardless of whether such has been traced.

Direct evidence to support the notion that progenitor cells can use more than one route to generate a type of mature cell comes from the experiments undertaken by Ishikawa and co-workers [15]. These workers purified CLP and CMP and derived dendritic cells (DC) *in vitro* from both the cell populations. The transcription profiles of the two DC populations were the same, supporting alternative lymphoid and myeloid routes to DC. Less direct support comes from examining the fates available to various progenitors and configuring the number of possible routes to an end cell type by virtue of which progenitors are or are not able to give rise to one another [reviewed in 3]. Using this approach, the known and possible routes for the development of neutrophils and monocytes are shown in Figure 2. For example, pathways to mature neutrophils include *via* LMPP, which have lymphoid in addition to myeloid potentials, and *via* CMP, which do not give rise to lymphocytes.

Some of the new models that challenged a lymphoid/myeloid dichotomy depict more than one route to an end cell type. In redefining lymphoid progenitors in 2002, Katsura mapped myeloid cells arising from a myeloid-T-cell progenitor and a myeloid-B-cell-progenitor in addition to from CMP [16]. Ye and Graf mapped two branches to granulocytes and monocytes, with one largely giving rise to granulocytes and monocytes and the second giving rise to these cells and platelets and erythroid cells [17]. An entirely new way of viewing flexibility of and reprogramming of cell lineages was presented in the ‘myeloid-based’ model by Kawamoto and Katsura [18]. These workers proposed HSC follow a ‘prototypic’ myeloid developmental pathway and these activities are then modified or switched off for HPC to undergo more specialised lymphoid development. Though the extent various routes are used *in vivo* remains uncertain, their existence supports the notion of lineage versatility.

**Figure 3:**
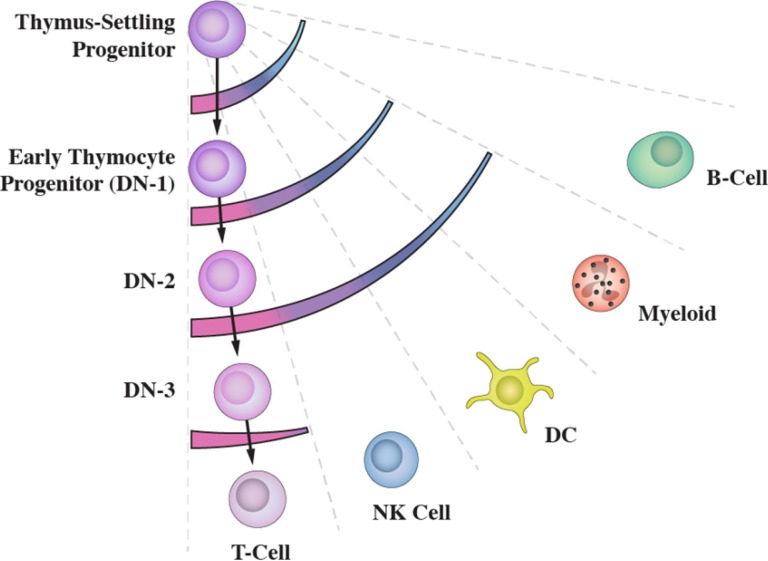
Developing thymus-settling progenitors retain clandestine options The sets of fate options available to thymus-settling progenitors as they differentiate towards T lymphocytes are shown by the arcs. They are revealed by culturing cells in appropriate conditions.

Commensurate with the notion of multiple routes is HPC that have developed some way along a pathway retain other fates as clandestine options. This is best illustrated by the development of progenitors from bone marrow that seed the thymus to undergo T cell development, termed thymus-settling progenitors (TSP). These cells can give rise to B lymphocytes, natural killer cells, myeloid cells and dendritic cells [19–22], as revealed by culturing cells in appropriate conditions. B lymphocyte potential is lost early during T cell development, as TSP become early thymocyte progenitors (double negative (DN) 1 cells). Cells at the DN1 and subsequent DN2 stages of development can still give rise to natural killer cells, myeloid cells and dendritic cells [19–21], with loss of these potentials as DN1 progress to the DN3 stage of T cell development (Figure 3). Whilst the extent to which developing TSP are diverted *in vivo* is uncertain these cells can clearly reprogram their fate.

### The importance of environmental signals to lineage programming

Not only are HSC and progenitors heterogeneous, but they also reside in a variety of microenvironments in which they interact with local stromal cells and receive signals from cytokines. These influences are now seen to be important to lineage programming of HSPC. The strength and duration of environmental signals, such as Notch, and presence of haematopoietic cytokines, such as macrophage colony-stimulating factor (MCS-F) and FLT3 ligand (FLT3L), influence the types of cells that, for example, developing thymocyte progenitors and EPLM give rise to *in vitro*. In early studies, whether signals consolidated the choice of lineage pathway or were instructive was unclear. Haematopoietic cytokines have long been viewed as providing signals for cell survival and proliferation. However, some are now clearly seen to instruct lineage fate decisions. Recently, Tsapogas and co-workers have shown that a certain threshold level of FLT3L drives multipotent progenitors towards myeloid/lymphoid fates at the expense of megakaryocyte/erythroid fates [22]. For many years, Metcalf championed instructive roles for granulocyte/macrophage colony stimulating factor (GM-CSF) and macrophage colony stimulating factor (M-CSF) in guiding granulocyte/macrophage colony forming cells towards granulocytes and macrophages, respectively [23]. In 1991, he showed a combination of GM-CSF or multi-colony stimulating factor, with stem cell factor, increased the frequency of granulocyte progenitors in blast cell colonies established from normal bone marrow cells [24]. In 2009, Reiger and co-workers confirmed instructive roles for M-CSF and G-CSF by showing these cytokines instruct individual GMP to adopt monocytic or neutrophilic fates, respectively [25]. M-CSF also instructs some long term-HSC (LT-HSC) to express myeloid associated genes [26]. Erythropoietin instructs erythroid fate as revealed by skewing of the potential of LT-HSC towards this fate and priming of erythroid lineage-associated genes in these cells [27].

As outlined above, the architecture of the haematopoietic system is far less compartmentalised and more complex, particularly in terms of lineage versatility, than previously thought. Developing HPC can use alternative pathways and have clandestine potentials, indicating that the natural propensities of HSPC are to diversify and versatility. This is in keeping with most of biology is Darwinian in nature. The presence of lineage biases within HSC might well extend to known HPC with each being a mixture of cells with differently biased differentiation capacities. Moreover, developing haematopoietic cells appear to be gradually biased towards producing one cell type or another which transforms our view of the nature of commitment in HSPC. In other words, lineage commitment isn't a stepwise all-or-none event, whereby pluripotent and/or oligo-potent populations of cells continuously produce committed offspring. And importantly, the biases can be overridden by environmental factors. All of the above have implications to the origins of and progression of malignant transformation. Below we examine whether transformed cells adhere to the above tendencies or have a more restricted phenotype overall.

### The architecture of leukemia: haematopoiesis and leukemia are both hierarchically organized processes

**Figure 4:**
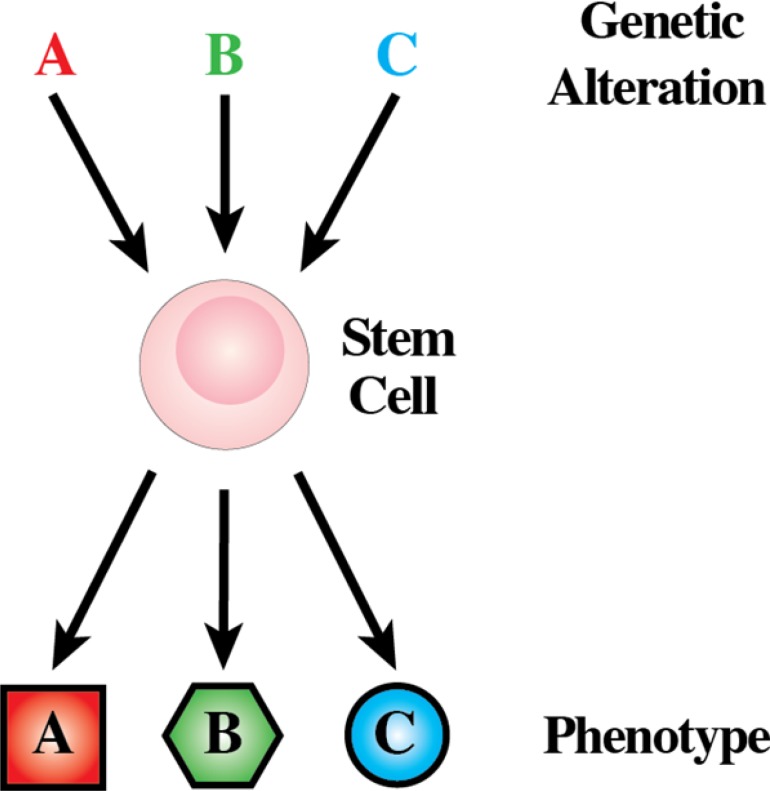
Genotype-phenotype correlations are established in human leukemias The expression of each one of the genetic lesions associates almost exclusively with a characteristic subgroup of human leukemias.

In the last years, we have seen the coming of age of the “leukemia stem cell” (LSC) theory. This has led to leukemia as a hierarchically organised process and renewed interest in whether aberrant cell differentiation lies at the root of cancer. From this point of view, a comprehensive knowledge of the aberrations to developmental mechanisms whereby normal target cells acquire this tumor characteristic is essential to understand leukemia [28]. Cytogenetic and molecular genetic analyses have defined genetic events that are consistently associated with each of the various types of cancer [29]. Particular genetic alterations are associated almost exclusively with a subgroup of human cancer that has a distinct phenotype (Figure 4). These genetic lesions are of clinical importance, and they may serve as unequivocal diagnostic markers, but they have also provided important clues to the cellular mechanisms behind leukemia development. The genotype-phenotype correlations established in human leukemias have demanded during the last three decades that we explain the nature of the intimate association between each genetic lesion and the phenotype (particular type of leukemia) with which it is associated [30].

**Figure 5:**
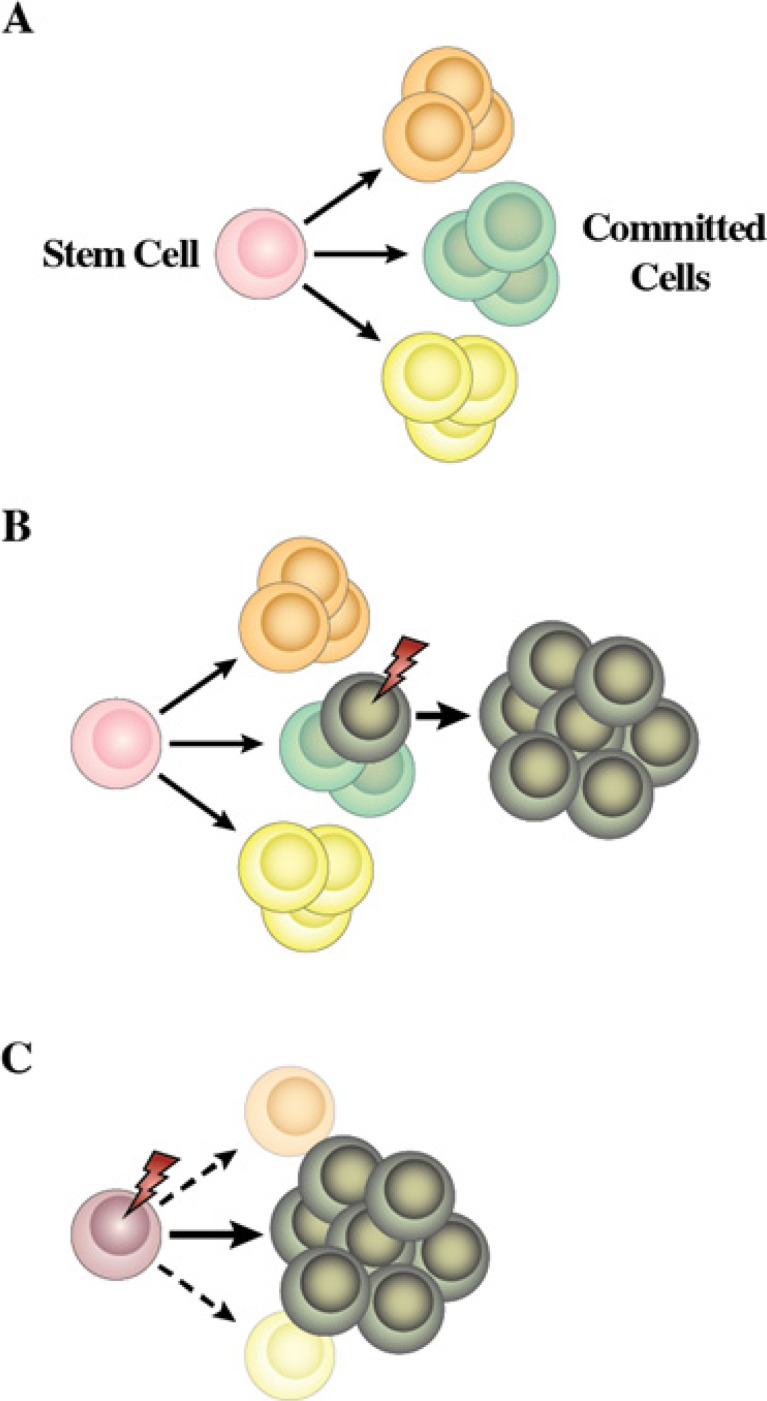
Proposed model for the role of human cancer gene defects in cell lineage specification **(A)** During haematopoiesis a small pool of multipotent stem cells maintain multiple cell lineages. **(B)** Traditionally, human leukemia genetic defects have been thought to act on cells already committed to a differentiation program. Hence, the leukemia phenotype closely resembles that of the initial differentiated target cell. **(C)** Normal uncommitted stem and progenitor cells are the targets for transformation in some human leukemias and the human leukemia-gene defects into these cells are the instigators of lineage choice decisions. Consistent with this is forced expression of these genes in stem cells can select or impose a specific leukemic-lineage outcome. This explains why specific gene defects are found only in one type of leukemia (see text for details).

Two different hypotheses have been considered to explain the link between genotype and the set phenotype of tumor cells (Figure 5). In the classical view of the initiation and progression of leukemia, an initiating genetic alteration takes place that is required for the immortalization of a committed/differentiated target cell (Figure 5B). Such cells will afterwards acquire additional genetic hits over time. The acquisition of additional hits aggravates the deregulation of the behavior of the differentiated target cell, leading to the clinically recognized features of leukemias. This is the model that has traditionally underpinned the study of leukemogenesis and takes for granted that the phenotype, in terms of the differentiated attributes of the tumor cells, reflects that of the normal cell that gave rise to the tumor in the first place. In most cases, cancerous cells do share similarities with non-pathological differentiated cell types. Therefore, for every kind of cancer, the cell of origin was assumed to be the corresponding normal differentiated cell. However and for some time, there have been some classical examples in which this is clearly not the case. Chronic myelogenous leukaemia (CML) presents as an abundance of relatively mature neutrophils and Fialkow and colleagues suggested nearly 40 years ago that this disease arises from transformation of a rare HSC. The evidence for this is the t (9;22) chromosomal translocation which typifies CML and can be found in most types of differentiated haematopoietic cells in patients [31]. During the chronic phase of CML the malignant stem cell clone is channelled towards producing neutrophils, and, in essence, the non-myeloid cells are not behaving in a malignant and clonally expansive manner. Another way of interpreting the genotype-phenotype correlations observed between genetic lesions and a given tumoral phenotype is to consider the possibility that the oncogene is directly responsible for imposing the specific characteristics of the leukemia phenotype (Figure 5C).

### Leukemogenesis: an inappropriate lineage-decision making process

There are striking findings that suggest that *tumoral reprogramming and aberrant lineage-programming* are key features of the origin of cancers and leukemias. Malignant glioblastoma neural stem cells can be reprogrammed to induced-pluripotent stem cells (iPSC). These iPSC can differentiate into mesodermal lineages, and when they do so they lose their malignant nature, but they maintain their malignant status when they differentiate along neural pathways [32]. Similarly, the differentiation block is overcome in primary human Philadelphia chromosome-positive B cell acute lymphoblastic leukemia (B-ALL) cells by reprogramming cells into non-leukemic macrophages [33]. The importance of these findings is that the malignancy status is somehow linked to the cells having been programmed to adopt a lineage pathway. Key questions are the developmental stage, as to stem and/or progenitor cells, which is being programmed to this effect and at what stage does this occur in relation to the progression of the development of leukemia.

During leukemogenesis a normal cell acquires a new but inappropriate (malignant) identity to give rise to a clonal aberrant population. This is only possible if the normal cell that gives rise to the leukemia, the leukemia cell-of-origin (LOC), has the necessary plasticity to undergo change. Also, the oncogenic event(s) initiating cancer must have an inherent reprogramming capacity, to be able to lead to the change in cellular identity [28]. The molecular mechanisms underlying this aberrant decision-making process are the pathological aberrations of the normal processes that regulate the developmental programming and plasticity of cells and that must operate to drive experimentally-induced reprogramming of mature cells to iPSCs. As to the capacity of an oncogenic insult to reprogram cells, it is generally accepted that tumoral progression is a multi-hit process. In this case different aspects of normal cellular biology are progressively altered to finally give rise to a full-blown tumor [34]. As mentioned above, developing normal HSPC are guided towards lineage biases, diversifying and differentiating towards their end-stage cells. The multi-hit requirement for full tumor development can be related to the notion that changes which are necessary for reprogramming cells to reverse a differentiated state to full pluripotency are inherently disfavoured developmentally. In both cases, biological barriers exist to prevent cells from changing their identity in this manner in order to avoid the risk of malignant transformation.

Evidence to support the inherent resistance of normal cells to reprograming by an oncogene to a tumour phenotype comes from recent studies of stem-cell based animal models of human cancer. Loss of p53 is a frequent occurrence in malignancy and facilitates pathological reprogramming to a malignant phenotype as follows. In a stem-cell based transgenic model of multiple myeloma, the loss of p53 accelerated the appearance of disease by allowing the *MafB* oncogene to drive a much more efficient malignant transformation [35, 36]. Something similar happens in the case of mucose-associated lymphoid tissue (MALT) lymphoma that is driven by the*MALT1* oncogene [37]. As to restoring activity of p53 protein and the effect on malignancy, a sophisticated approach has been the use of a knock-in mouse model carrying the modified p53 protein p53ERTAM. This can be switched on and off *in vivo* by tamoxifen administration or withdrawal, respectively. In a stem-cell based model of CML [38], restoration of p53 activity slowed the progression of the disease and extended the survival of leukemic animals by inducing the apoptotic death of primitive leukemic cells.

The relevance of reprogramming and lineage decision-making to malignancy is also illustrated by consideration of the action of retinoic acid receptors (RARs), particularly RARγ. These receptors, for the natural ligand all-*trans* retinoic acid, have long been known to play important roles during development. The oncogenic potential of RARγ was revealed by studies of hepatocellular carcinoma [39]. As to a link to lineage reprogramming, addition of RARγ to the four transcription factors (Oct4, Sox2, Klf4 and c-Myc) used to reprogram somatic cells to iPSCs cells greatly accelerated reprogramming of mouse embryonic fibroblasts [40]. The importance of activity of RARγ to decision-making by stem cells is seen in studies of zebrafish development. Treatment of zebrafish embryos with a RARγ-specific agonist adversely affected the development and growth of tissues that form from neural crest and lateral plate mesodermal stem and progenitor cells that express RARγ. These studies revealed that RARγ, which can play a role in cell reprogramming, has to be inactive at the stem/progenitor stage of development for these cells to make an appropriate decision to differentiate along a pathway [41].

### The cell of origin in leukemia

**Figure 6:**
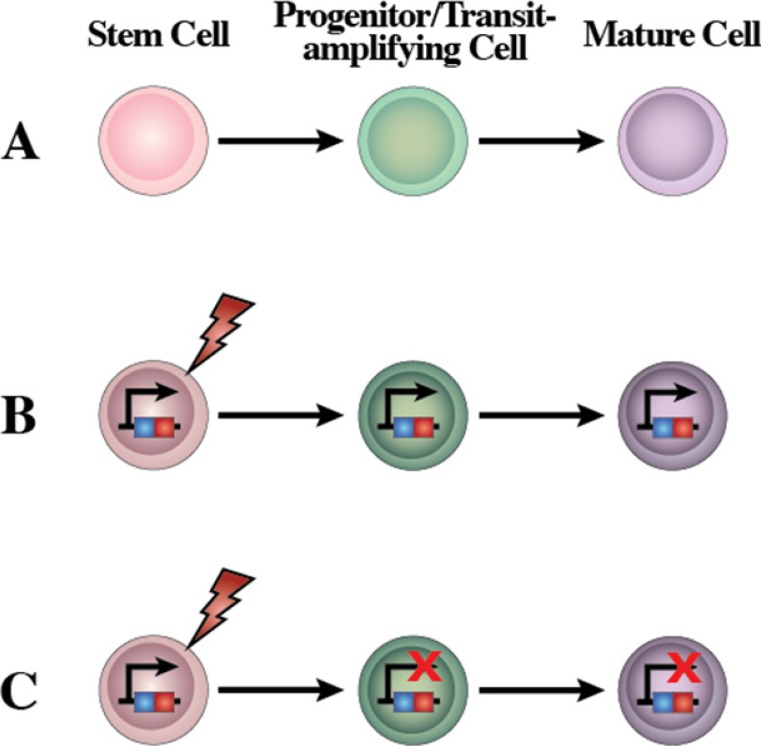
A new concept of the human leukemia as a result of a restriction of lineage options during stem cell transformation **(A)** Scheme of the normal differentiation program from stem cells. Normal stem cells give rise to transit cells which expand to give rise to terminally differentiated cells. **(B)** Human leukemia is a genetic disease originated by several possible types of genetic/epigenetic alterations. LSC give rise to transit-amplifying cells that would expand and originate the main and highly expansive tumor cell mass. All human leukemic cells carry the oncogenic alteration, from the cell-of-origin to the more differentiated cancer cells, though the role of this oncogene may be different at different stages of leukemia differentiation, and these mutations might become carrier mutations rather than driving ones depending on the cellular context. **(C)** Based on the reprogramming nature of oncogenes, restricting expression of the oncogenic alterations to the stem cell compartment is all that is needed to recapitulate the heterogeneity of leukemia. Using a stem-cell restricted transgenic expression system, the expression of the oncogene in the reprogramming-prone stem cells and progenitors allows the development of all of the cells that compose the leukemia mass. The modified gene is present in all the mouse cells but the oncogene impact is limited to the stem/progenitor compartment. This is similar to what happens in other cases of reprogramming, where the reprogramming factor(s) does not need to be present anymore once the initial fate-inducing change has taken place (for example, induced pluripotency).

Accepting that leukemia is a hierarchically organized tissue maintained by leukemia stem cells (LSCs), it becomes essential to identify the normal cell which first suffers the oncogenic alteration that will finally lead to the generation of the LSC (Figure 6). This LCO must possess sufficient plasticity to allow the tumoral reprogramming to take place or, at least, to be initiated. The characteristics of the final leukemia will be the result of the interaction between the reprogramming capacity of the oncogene(s) and the susceptibility of the LCO to reprogramming. As mentioned above, it was assumed that the phenotype of the leukemia cells reflects that of the normal cell from which they originated [30], and that, therefore, the characteristics of leukemia cells were more or less a caricature of their cell of origin. Accordingly, the cell of origin was always sought among those most similar to the cancerous one. However, all the evidence accumulated over the last years has led to a different point of view that leukemias are a stem cell-based tissue [42–44]. In this case, only some of the cells within the tumor, namely the LSC can regenerate the leukemia mass and the phenotype of the tumoral differentiated and partially differentiated cells might be largely unrelated to the cell that originates the LSCs. The LCO would be the cell by the process of tumoral reprogramming that will finally lead to the generation of the LSC which is the only one that can (re)generate the tumor.

One option is that LSCs could have their origin in normal stem cells, maintain stem cell properties and become pathologically reprogrammed *via* a mutational event (Figure 6). In the case of CML, the tumor-driving mutation is the chimeric oncogene *BCR-ABLp210*. It has long been known that *BCR-ABLp210* is present in all the hematopoietic lineages and can also be traced back to their HSCs. Importantly, the oncogenic effect only manifests in the myeloid lineages, at least during the chronic phase [45]. The stem cell origin of CML has been recently confirmed using a stem-cell based animal model in which expression of the *BCR-ABLp210* oncogene is restricted to the stem cell stage of development in a genetically engineered mouse [43, 46]. Despite this restricted expression window, *BCR-ABLp210* induces a full-blown CML in the mice, with all the cellular and tissue characteristics of the human disease including cells differentiating towards neutrophils. Importantly, the differentiated CML cells do not express the oncogene but have a tumoral phenotype, therefore indicating that tumoral reprogramming took place at an earlier developmental level [43, 46]. Similarly, in transgenic mice in which the expression of different oncogenes is restricted to the stem/progenitor cells, mice develop specific leukemias/lymphomas that very closely recapitulate the main features of human disease [36, 37, 46–48]. However, heterogeneity seems to be restricted to the initial stages of chronic leukemia and tumors and importantly sub-clones mainly determine tumor progression and therapeutic outcomes [49, 50].

Further support to the notion that leukemias largely arise from stem cells is their longevity predisposes to malignant transformation. Recent advanced technologies including next-generation sequencing analysis have enabled us to identify frequently occurring somatic mutations in many hematological malignancies including leukemias and lymphomas [51–69]. It is important to note that only cells with a long lifespan can accumulate these mutations. In regard to haematopoiesis, the HSCs are the main critical cellular targets and it is known that the number of acquired somatic mutations in HSC increase with age [70]. HSCs carrying these mutations can produce a number of myeloid or lymphoid progenitors that have identical abnormalities and these progenitors can be the final target for the transformation of LOCs into LSCs in acute leukemia [71]. As outlined above, the *BCR-ABLp210* fusion protein can drive HSCs to give rise to the chronic phase of CML. However, during myeloid blast crisis in CML, additional genetic abnormalities can transform GMP into LSCs. In *t* (8; 21) AML, the AML1–ETO fusion is found in HSCs; these AML1–ETO^+^ HSCs can differentiate into mature blood cells [72, 73]. Additional KIT mutations at the GMP stage seem to be critical for the formation of AML LSCs [74]. Furthermore, next-generation sequencing analyses have shown that pre-malignant clones carrying somatic mutations have been frequently found in HSCs of patients with AML without specific chromosomal abnormalities [75, 76]. Thus, HSCs are a reservoir for mutations at least in myeloid malignancies [77].

In contrast, the involvement of HSCs had not been considered in lymphoid malignancies within the recent past. However, it has been reported recently that in mature lymphoid malignancies, such as chronic lymphocytic leukemia (CLL), the cellular propensity for clonal B cell development has been achieved at the HSC level [78, 79]. And, the genetic alterations specific for T cell lymphoma [80, 81], follicular lymphoma [82, 83] and hairy cell leukemia [84] can be traced back to the HSC stage. These studies have suggested that even in relatively mature lymphoid malignancies, human HSCs could be the reservoir for genetic mutations, which constitutes a prime source for lymphoid malignancy development. The outcomes are cells that are veered towards particular developmental pathways. In addition to the detailed analysis of LCO in primary human lymphoid malignancies, murine models of mature B-cell malignancies have been successfully established by targeting HSCs; the ectopic expression of disease-specific oncogenes such as *Braf* V600E, *MALT1*, *MafB*, *HGAL*, and *Bcl6* within HSCs successfully reproduces the nature of the mature lymphoid malignancies [36, 37, 47, 48, 84]. Importantly, when these oncogenes have been expressed within cells committed to the B-cell lineage the mice failed to develop the lymphoid malignancy. These studies support the hypothesis that mature lymphoid malignancies may be initiated by an inappropriate lineage-decision making process at the HSC level.

### Restriction of lineage options during stem cell transformation

**Figure 7:**
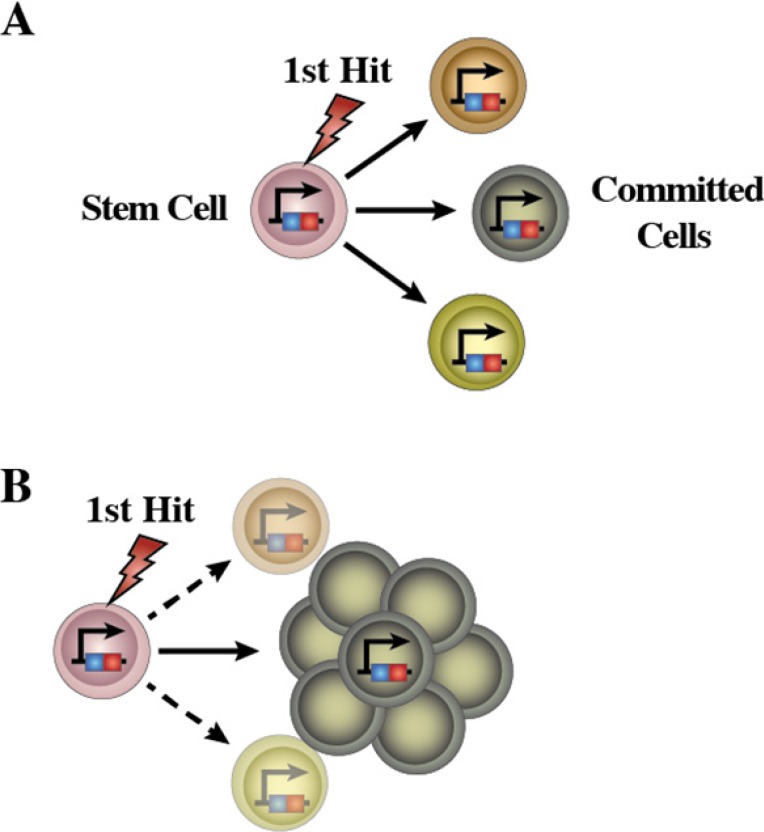
Schematic representation of the emergence of LSCs in human leukemia **(A)** A mutation occurs in HSCs leading to the emergence of aberrant pre-leukemic HSCs. These aberrant pre-leukemic HSCs self-renew and expand within the HSC compartment. Pre-leukemic HSCs give rise to a high number of lineage-committed progenitors harboring the identical mutations. This leads to an increased chance of acquiring the additional oncogenic events, which finally transform the aberrant progenitor cells from pre-leukemic HSCs into the leukemic stem cells (LSCs). **(B)** Loss of differentiation potentials is essential for the emergence of LSCs. These sequential leukemia progression models are commonly accepted as to the development process of human acute malignancies.

Normal HSCs are characterized by their multilineage differentiation potential. The pre-LSCs of many leukemias exhibit multilineage potential and differentiation, as mentioned above, pre-LSC CML associated with *BCR-ABL* fusion exhibit multilineage differentiation whereby mature granulocytes, monocytes, erythrocytes, platelets and B lymphocytes are all part of the malignant clone (Figure 7A). Similarly, in CML associated with a mutant *RAS* allele, the mutant *RAS* can be found in granulocytes, monocytes, B lymphocytes and T lymphocytes, indicating also the presence of a multipotent pre-LSC. In myelodysplastic syndromes, a multipotent malignant stem cell is implicated in patients with refractory anemia (RA), RA with ringed sideroblasts or RA with excess blasts. The presence of pre-LSC with multilineage differentiation potential suggests the occurrence of initiating mutations in a normal HSC. Only upon the acquisition of further mutations the initiated LCO evolves to produce a sub-clone of lineage-restricted malignant blasts (Figure 7B). This fits with a two-hit model of leukemogenesis, which posits the stepwise acquisition and collaboration between mutations that activate signal-transduction pathways to confer a survival or proliferative advantage (e.g. mutations in *FLT3*, *RAS* or *KIT*) and mutations in genes coding for transcriptional regulators that potentially restrict lineage options (e.g. generation of novel oncogenes such as *RUNX1-RUNX1T1* or *PML-RARA*, *BCR-ABLp190, ETV6-RUNX1* or mutations in *CEBPA*, *PAX5* or *NPM1*). In keeping with this are cases in which a pre-leukemia lesion exists stably as a single aberration in an abnormal cell population that will only progress to an open leukemia when secondary hits occur.

### Targeted therapies fail to eradicate LSC and the prospect of reprogramming these cells

Current therapies fail to eradicate LSC, in spite of their apparent efficacy against the main mass of leukemia cells. A possible rationale to this difficulty is as follows. Above we have argued that LSC are generated by a (leukemia) reprogramming of normal HSC to a set (leukemic) phenotype whereby lineage options have been restricted. In this case, the triggering agents, the oncogenes that initiate leukemia formation, might not be required anymore for disease progression once the new circuits have been established and the new fate is set [46, 85]. The initiating lesion is the driving force in the reprogramming process, essential for leukemogenesis. However, once reprogramming and lineage restriction have taken place the initiating hit is only a passenger mutation within the LSC, either without a significant function anymore or even performing a different role, unrelated to the reprogramming one, in tumor expansion or proliferation. This model can explain why oncogene inhibition through targeted therapies would not affect the LSC compartment.

As a driver to leukemia, reprogramming of HSPC to HSC and to a distinct lineage option circuitry presumably leads to the establishment of a new epigenetic signature. The latter opens a clear hope for treatment as epigenetic modifications, unlike genetic changes, can be manipulated to erase and/or reverse. Indeed, it has already been proven that incorporation of agents that target epigenetic events to the standard chemotherapy is a promising approach to the treatment of relapsed pediatric acute lymphoblastic leukemia [86]. In different experimental settings, the results suggest that cancer cells can be reprogrammed to a non-tumoral fate, losing their malignancy. For example, it is possible to even produce clonal mouse embryos from brain tumors [87] and, by using nuclear transplantation, to reprogram melanoma cells [88] and embryonal carcinomas [89]. As described above, it is clear that reprogramming B-ALL cells to an alternative lineage cell fate can suppress malignancy. These findings indicate that reprogramming tumor cells is a viable prospect. Also, it is to be expected that LSCs from different cancer types will share many similarities, implying that similar LSC-based therapeutic approaches could be used in many different leukemias. However, as for any other therapeutic approach, a precise knowledge of the epigenetic rewiring is necessary before we can attempt a successful intervention.

### Future opportunities and challenges

For the future, there is a biological paradox we don't really understand, in particular the balance between cell intrinsic and environmental drivers of certain biological processes. Pluripotency, a pre-requisite for versatility, underpins the means of an organism to diversify by evolving different types of specialized cells. However, maintenance of pluripotency is obstructive to the embryonic development of a specialized adult organism from the amorphous mass of cells that is the early embryo. We are moving towards the viewpoint that the architecture of normal haematopoiesis is much more versatile and plastic than previously thought. These attributes are important to responding to the demand provoked by a wide variety of infectious agents as to the specialized cells required to counteract such. We have postulated leukemias, in their full-blown and overt state, are channeled, *via* reprogramming, towards a malignant and more immobile phenotype. Do we see that cancer is the price to pay as to exacerbation of an inherent and selfish-gene propensity to enforce lineage biases which is important to maintenance of the overall *status quo* of the developed adult organism? This ancestral biological drive exists as some organisms have remained unchanged for millions of years.
